# Circulating Exosome microRNAs as Diagnostic Biomarkers of Dementia

**DOI:** 10.3389/fnagi.2020.580199

**Published:** 2020-09-08

**Authors:** Xiaoyu Dong, Dongming Zheng, Jianfei Nao

**Affiliations:** Department of Neurology, Shengjing Hospital of China Medical University, Shenyang, China

**Keywords:** exosome miRNA, dementia, cerebrospinal fluid, blood, biomarker

## Abstract

Dementia is a syndrome of acquired cognitive impairment that leads to a significant decline in a patient’s daily life, ability to learn, and the ability to communicate with others. Dementia occurs in many diseases, including Alzheimer’s disease (AD), dementia with Lewy bodies, frontotemporal dementia, and Parkinson’s disease dementia (PDD). Although the analysis of biomarkers in the cerebrospinal fluid (CSF) and peripheral blood physicochemical analysis can indicate neurological impairment, there are currently no sensitive biomarkers for early clinical diagnosis of dementia or for identifying the cause of dementia. Previous studies have suggested that circulating micro (mi)RNAs may be used as biomarkers for diagnosing neurological disorders. However, miRNAs are susceptible to interference by other components in the peripheral circulation, bringing into question the diagnostic value of circulating miRNAs. Exosomes secreted by most cell types contain proteins, mRNAs, and miRNAs that are closely associated with changes in cellular functions. Exosome miRNAs (ex-miRNAs) are highly stable and resistant to degradation. Therefore, these may serve as useful biomarkers for the early clinical diagnosis of dementia. Here, we review studies of ex-miRNAs that commonly cause clinical dementia and explore whether ex-miRNAs may be used as early diagnostic biomarkers of dementia.

## Introduction

Dementia is a syndrome of acquired cognitive impairment. It leads to a significant decline in a patient’s ability to live, study, work, and socially interact with others (Sacuiu, 2016; Gale et al., 2018). Many diseases cause dementia, including Alzheimer’s disease (AD), which accounts for 50% to 70% of cases; vascular dementia (VD), which accounts for 15% to 20% of cases; dementia with Lewy bodies (DLB, 5% to 10% of cases); frontotemporal dementia (5% to 10% of cases); and Parkinson’s disease dementia (PDD), which accounts for about 3.6% of cases (Writing Group of the Dementia and Cognitive Society of Neurology Committee of Chinese Medical Association; Alzheimer’s Disease Chinese, 2010). As global aging continues to increase, the number of dementia patients increases each year (Maloney and Lahiri, 2016). For example, there are more than 30 million AD patients worldwide. The incidence of AD is sporadic, and only 5% of patients have early-onset dementia related to genetic causes (Hung et al., 2016). The World Health Organization estimates that by 2050, there will be 115 million AD patients worldwide. Zeng et al. reported in the Disease Burden Report of China from 1990 to 2016 that China’s AD growth rate from 2005 to 2016 was 57.8%. However, it is difficult to detect protein misfolding and/or neurodegenerative processes before the onset of cognitive decline.

In recent years, following rapid advancements in diagnostic technology, considerable progress has been made in the study of biochemical diagnostic biomarkers for dementia (Herukka et al., 2017; Simonsen et al., 2017; Dos Santos Picanco et al., 2018). Several studies have suggested that the levels of plasma interleukin (IL)-10 and IL-6 in the peripheral blood and cerebrospinal fluid (CSF) are useful for clinical diagnosis of AD (Asselineau et al., 2015; Gaur and Agnihotri, 2015; Baiardi et al., 2018; Yang C. C. et al., 2018). However, their effectiveness as biomarkers has been questioned. Also, as CSF biomarker analysis can be carried out with automated chemiluminescent platforms with high reproducibility and minimal effort, CSF biomarkers like amyloid β-peptide (Aβ)42/Aβ40, p-181-tau, and total tau have been used to identify AD independent of the clinical-stage (Jack Jr et al., 2018). However, novel biomarkers in peripheral fluids, such as plasma or serum, could facilitate early diagnosis and limit patient discomfort compared to a lumbar puncture. Factors related to oxidative stress, including malondialdehyde, reactive oxygen species, 8-hydroxy-2-deoxyguanosine, platelets, ubidecarenone, uric acid, and glutathione have been detected at various levels in the peripheral fluids of patients with PD (Bolner et al., 2016; Islam, 2017; Pandey et al., 2018). The mean level of urinary 8-hydroxy-2-deoxyguanosine increases as PD progresses and is not affected by dopamine. Therefore, 8-hydroxy-2-deoxyguanosine may be a useful biomarker for evaluating the progression of PD (Sato et al., 2005). Additionally, it has been suggested that the detection of CSF α-synuclein in PD patients may be a promising definitive biomarker for PD diagnosis. Gao et al. (2015) performed a systematic study of all relevant research studies investigating reproducible CSF α-synuclein quantification methods. They found that the mean CSF α-synuclein concentration was significantly lower in PD patients compared to controls, and there was no significant difference in mean CSF α-synuclein between PD patients and DLB patients. They concluded that detection of α-synuclein alone was not sufficient as a single biomarker and that it must, therefore, be measured in conjunction with other documented and reliable biomarkers to diagnose PD and differentiate synucleinopathies (Gao et al., 2015). Studies have suggested that the protein-misfolding cyclic amplification (PMCA) and real-time quaking-induced conversion (RT-QuIC) assays may be useful for the diagnosis of synucleinopathies, especially PD. However, several issues need to be addressed to increase the utility of these assays, including standardizing operating procedures, decreasing the duration of the assays, identifying “false positions,” and increasing their sensitivity (Paciotti et al., 2018; Kang et al., 2019). Therefore, the detection of novel biomarkers, in conjunction with CSF α-synuclein detection, in peripheral fluids is of great clinical value for the diagnosis of PD and differentiation of synucleinopathies.

MicroRNAs are a type of endogenous non-protein–encoded single-chain short-sequence RNA which mainly regulate cellular protein expression at the post-transcriptional level and participate in cell development, differentiation, proliferation, and apoptosis (Tafrihi and Hasheminasab, 2019). MicroRNAs play important roles in a variety of physiological functions, including nervous system morphogenesis, synaptic plasticity, and memory (Lee et al., 2011; Molasy et al., 2017). Circulating miRNA is easy to obtain from plasma, serum, CSF, urine, and saliva; it is easy to detect and is tissue-specific (Foye et al., 2017). Because of this, it has been suggested that circulating miRNAs may be useful biomarkers for diagnosis, monitoring of drug efficacy, and prognostic evaluation of neurological diseases. Chopra et al. (2020) suggested that dysregulation of miRNA-298, which down-regulates the expression of amyloid precursor protein (APP) and beta-secretase 1 mRNAs, may play important roles in the incidence and progression of AD, especially since changes in miRNA-298 levels in the blood and CSF of AD patients have been observed. Levels of miRNAs may differ in preclinical conditions and may be considered diagnostic biomarkers for AD (Hajjari et al., 2017). Müller et al. (2016) found differential expression of miRNA-29a, miRNA-125b, and miRNA-146a in the CSF of patients with AD compared to healthy controls, indicating the potential diagnostic value of circulating miRNAs. Li et al. (2017) reported that plasma levels of miRNA-137 were significantly increased in patients with PD while miRNA-124 levels were decreased, and these assays had high sensitivity and specificity for PD. However, miRNAs in the peripheral circulation are susceptible to interference from other blood components, resulting in inconsistent results and limited application of miRNAs as clinical diagnostic biomarkers.

Exosomes are tiny membrane vesicles that can be secreted by most cells in the body. They have a lipid bilayer membrane and are about 30–150 nm in diameter (Graner, 2019). Exosomes are present in various bodily fluids and carry lipids, proteins, DNA, miRNAs, mRNAs, long non-coding RNAs, and other genetic material. Exosome (ex)-miRNAs may be protected from degradation by nucleases widely present in circulating fluids (Feng et al., 2018). Therefore, detection of specific miRNAs in exosomes may be used for disease diagnosis. CSF is in direct contact with the central nervous system (CNS) and accurately reflects any biochemical changes in the CNS. However, obtaining CSF through lumbar puncture is invasive and comes with an increased risk of intracranial infection. Ex-miRNAs are stable in the blood and can be reliably detected at low concentrations (Coenen-Stass et al., 2019). Therefore, the study of ex-miRNAs in the blood may provide promising early diagnostics for dementia patients.

Several studies have reported that ex-miRNAs are involved in the pathogenesis of dementia (Sierksma et al., 2018; Chen et al., 2019). A recent microarray study of salivary ex-miRNAs from young and old healthy subjects performed by Machida et al. (2015) identified ex-miRNA-24-3p as a possible peripheral aging biomarker. Sarkar et al. (2016) found that the expression of miRNA-34a was up-regulated in the temporal lobe of AD mice and patients. After highly expressed miRNA-34a was taken up by neighboring neurons, a disintegrin, metalloprotease (ADAM) 10, and silent information regulator factor 2-related enzyme 1 were down-regulated and Aβ was deposited, suggesting that abnormally expressed miRNAs are involved in AD pathogenesis (Sarkar et al., 2016). Elevated levels of miRNA-126 may play a functional role in AD neurons and contribute to the incidence of PD by down-regulating insulin-like growth factor/phosphoinositide 3-kinase signaling (Kim et al., 2014). Chen-Plotkin et al. (2012) found that three members of the miRNA-132 cluster were significantly down-regulated in frontotemporal dementia (FTD) brains with Tar DNA-binding protein 43 inclusions. This resulted in the upregulation of transmembrane protein 106B, which was a risk factor for FTD, suggesting that dysregulated miRNAs may contribute to the pathogenesis of this disease (Chen-Plotkin et al., 2012). In addition to participating in the pathogenesis of dementia, circulating ex-miRNAs may also be promising biomarkers for its diagnosis. In this review, by briefly describing the physiological characteristics of ex-miRNAs and reviewing studies of ex-miRNAs that commonly cause clinical dementia diseases, we explore whether ex-miRNAs may be used as early diagnostic biomarkers of dementia.

## Synthesis and Packaging of miRNAs Into Exosomes

Genes encoding miRNAs in the nucleus are transcribed by RNA polymerase II to produce primary transcripts (pri-miRNAs). pri-miRNAs are similar in structure to protein-encoding mRNAs and contain 3’ polyadenylation and 5’ end caps (Stavast and Erkeland, 2019; Seelan et al., 2020). The Drosha and Drosha-associated binding protein Pasha (also known as DGCR8) complex cleave pri-miRNAs to form miRNA precursors (pre-miRNAs) with a stem-loop structure of about 70 nucleotides in length (Jin et al., 2020). Pre-miRNAs are transported from the nucleus to the cytoplasm through the action of the Ran-GTP–dependent cytoplasmic/cytoplasmic transport protein exportin 5 (Pan et al., 2019). The Dicer enzyme then cleaves the miRNA precursor into double-stranded miRNAs, which are 21–25 nucleotides in length. Two free nucleotides are present at the 3’ end of both strands of miRNA (Chen et al., 2008). Initially, the mature miRNA and its complementary sequence form a double helix structure. Subsequently, the double helix structure unwinds, and a mature single strand binds to the RNA-induced silencing complex (RISC) in an asymmetric manner (Wang et al., 2013). The complex binds to mRNAs with complementary sequences and regulates gene expression by modulating the stability of the transcripts or the translation of target mRNAs.

Exosomes are cystic structures secreted by a variety of cells that function in material transport, information transmission, and antigen presentation. Exosomes contain a large number of components, such as mRNA, miRNA, circular RNA, and DNA fragments. These contents provide copious amounts of biological information that can potentially reveal information about disease status at multiple levels (Koritzinsky et al., 2017; Zhang et al., 2020). The packaging of miRNAs into exosomes in the cytoplasm is regulated by a variety of mechanisms, including a neuro-phospholipase-dependent pathway, a heterogeneous ribonucleoprotein-dependent pathway, the 3’-end sequence-dependent pathway of miRNAs, and the miRISC-related pathways (Higa et al., 2014; Batool et al., 2020). It has been suggested that ex-miRNAs from the CNS may reflect its physiological status and provide a reference for the evaluation of dementia (Zagrean et al., 2018).

The isolation and purification of exosomes are mainly based on their size, density, and biochemical characteristics. The most common techniques for isolating exosomes include removing cell debris by layered centrifugation and then purifying exosomes by ultracentrifugation to eliminate contaminating proteins (Gurunathan et al., 2019). Based on the specific density of exosomes, which can be separated and purified by sucrose density gradient separation, ultrafiltration, and chromatographic analysis are commonly used separation and purification techniques (Jeppesen et al., 2019). Newly developed fluorescent label-based high-resolution flow cytometry has obvious advantages for the qualitative and quantitative analysis of exosomes (Haraszti et al., 2016). This technology not only allows for the high throughput measurement of single vesicles and multiple parameters but also enables the detection of vesicle surface-specific proteins. This is a feature of great significance for the identification of exosome subtypes.

**Figure 1:**
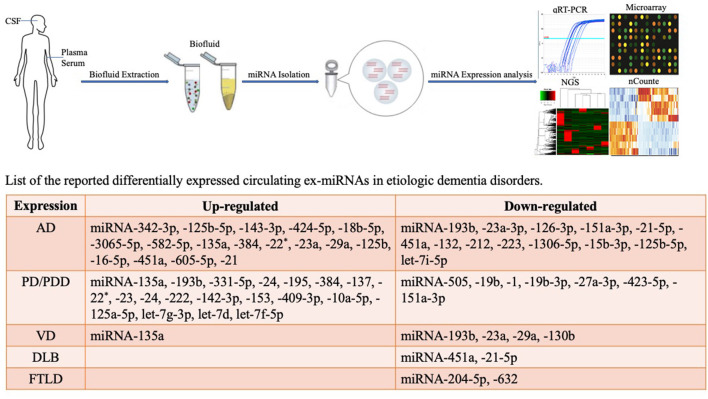
Methodological aspects of circulating biomarker research and circulating exosome miRNAs as biomarkers for dementia disorders.

This review focuses on changes in exosome miRNAs in the CNS under pathological conditions and provides a reference for diagnostic biomarkers for the characterization of changes in the physiological status of dementia (Figure 1).

## Circulating Ex-miRNAs as Biomarkers for Dementia

Dementia disorders usually have a long incubation period before the onset of clinical symptoms. Current clinical treatment strategies are mainly aimed at improving symptoms and delaying further development of the disease. For these reasons, early diagnosis of dementia is extremely important; identification of stable and sensitive biomarkers of disease progression is particularly critical. Ideal dementia diagnostic biomarkers should effectively indicate neurodegenerative changes in the brain before the occurrence of cognitive impairment and show specificity, predictability, and operability (Rossor et al., 2010; Sun et al., 2019; Zhang et al., 2019). Because of the important regulatory roles of miRNAs in dementia, peripheral blood miRNAs are expected to become new biomarkers of dementia (Hoss et al., 2016; Yao et al., 2019). MicroRNAs transported by exosomes may improve diagnostic accuracy, since they completely maintain neuronal information during secretion. Therefore, ex-miRNAs have potential value as diagnostic biomarkers for dementia (Van Giau and An, 2016).

## Ex-miRNAs as Biomarkers in AD

Existing studies have isolated exosomes from the plasma of patients with AD, analyzed the expression of miRNAs with high-throughput sequencing technology, and then compared the results with those of a control group. One clinical study enrolled 35 AD patients and 35 healthy controls to assess the value of ex-miRNAs as biomarkers for AD. Twenty miRNAs showed significant differences in the AD group. Of these, miRNA-342-3p was significantly lower in patients with AD (Lugli et al., 2015). By comparing differently expressed miRNAs between 150 AD patients and 150 healthy controls, Tan et al. (2014) also found that miRNA-342-3p was dysregulated in AD patients with high sensitivity and specificity (81.5% and 70.1%, respectively). The expression of miRNA-125a-5p, miRNA-125b-5p, and miRNA-342-3p was found to be up-regulated in the brains of patients with AD (Hinske et al., 2014). In a study by Rani et al. (2017), plasma levels of ex-miRNAs were analyzed in 97 AD patients. They found that ex-miRNA-342-3p and ex-miRNA-125b-5p were increased and exhibited strong correlations (*R*^2^ = 0.09 and 0.092, respectively) with the patients’ Montreal Cognitive Assessment scores (Rani et al., 2017). In another study, researchers found that miRNA-193b could affect the progression of AD by targeting and regulating APP in AD mice. When comparing the ex-miRNA expression profiles of 43 mild cognitive impairment (MCI) and 51 AD patients, Liu et al. (2014) found that ex-miRNA-193b was significantly lower in MCI and AD patients than in the control group. Also, the expression of ex-miRNA-193b was lower in patients with AD than in patients with MCI. ex-miRNA-193b expression also negatively correlated with Aβ42 expression. This study showed that ex-miRNA-193b could be used as a potential diagnostic biomarker to reflect the progression of AD (Liu et al., 2014). Gámez-Valero et al. (2019) enrolled 10 patients with AD, 11 patients with DLB, and 11 age-matched controls and analyzed differentially expressed ex-miRNAs in these groups. Plasma levels of ex-miRNA-23a-3p, ex-miRNA-126-3p, ex-miRNA-let-7i-5p, and ex-miRNA-151a-3p were found to be significantly decreased in AD compared to healthy controls. Ex–miRNA-21-5p and ex-miRNA-451a were decreased in patients with AD compared to patients with DLB and healthy controls. Moreover, ex-miRNA-451a (AUC = 0.95) and ex-miRNA-21-5p (AUC = 0.93) showed predictive diagnostic value, based on receiver operating characteristic (ROC) curves, in discriminating between patients with AD or DLB with high specificity and sensitivity (Gámez-Valero et al., 2019). Plasma ex-miRNA-132 and ex-miRNA-212 were also decreased in patients with AD compared to healthy controls. Cha et al. (2019) analyzed the differences in miRNAs in brain extracts from 11 AD patients, seven high pathological controls (HPC), and nine controls. They suggest that the measurement of ex-miRNA-132 (AUC = 0.77) and ex-miRNA-212 (AUC = 0.84) in plasma could be further investigated as diagnostic aids for AD and as potential therapeutic targets (Cha et al., 2019).

Wei et al. (2018) examined serum ex-miRNA-137, ex-miRNA-155, and ex-miRNA-223 levels in 32 dementia patients and compared these to the levels in 16 healthy controls. They found that ex-miRNA-223 levels were significantly decreased in dementia patients and markedly correlated with the severity of cognitive impairment and serum concentrations of neuroinflammatory factors. This suggests that serum ex-miRNA-223 (AUC = 0.875) may be a promising biomarker for diagnosing dementia and evaluating the progression of the disease (Wei et al., 2018). Cheng et al. (2015) explored the differential expression of serum ex-miRNAs between 23 patients with AD, three patients with MCI, and 23 healthy controls. Five upregulated ex-miRNAs (miRNA-143-3p, miRNA-424-5p, miRNA-18b-5p, miRNA-3065-5p, and miRNA-582-5p) and two downregulated ex-miRNAs (miRNA-1306-5p and miRNA-15b-3p) were identified to have diagnostic value for dementia (Cheng et al., 2015). Yang T. T. et al. (2018) evaluated the potential value of serum ex-miRNA-135a, ex-miRNA-193b, and ex-miRNA-384 as biomarkers for the diagnosis of AD. They found that ex-miRNA-135a and ex-miRNA-384 were upregulated, while ex-miRNA-193b was downregulated in patients with AD. While ex-miRNA-135a and ex-miRNA-193b were both significantly increased in patients with PDD, the former had a marked diagnostic value for such patients (Yang T. T. et al., 2018). By mining the literature, Barbagallo et al. (2020) identified 23 neurodegenerative disease-associated miRNAs and investigated serum expression in a cohort of 139 patients, including in patients with AD (*n* = 30), PD (*n* = 30), VD (*n* = 24), vascular parkinsonism (VP; *n* = 25), and healthy controls (*n* = 30). They found that ex-miRNA-22*, ex-miRNA-23a, ex-miRNA-29a, and ex-miRNA-125b levels were increased in the serum of patients with AD compared to healthy controls (AUC = 0.71), suggesting that measurement of ex-miRNA levels could identify dementia disorders in a non-invasive manner, thus improving clinical diagnosis (Barbagallo et al., 2020).

A CSF miRNA profile study for the diagnosis of AD was performed by Riancho et al. (2017). Their study, which had enrolled 10 patients with AD and 10 healthy controls, suggested CSF ex-miRNA-9-5p and ex-miRNA-598 could be potential biomarkers for AD (Riancho et al., 2017). Differentially expressed CSF ex-miRNAs also have a diagnostic value for early- and late-onset AD. In a study by Mckeever et al. (2018), 17 young-onset AD (YOAD) and 13 late-onset AD (LOAD) patients were enrolled. The levels of ex-miRNA-16-5p (AUC = 0.76), ex-miRNA-451a (AUC = 0.95), and ex-miRNA-605-5p (AUC = 0.71) were found to be significantly increased, while the ex-miRNA-125b-5p level was decreased (AUC = 0.72) in YOAD patients. The levels of ex-miRNA-125b-5p (AUC = 0.79), ex-miRNA-451a (AUC = 0.85), and ex-miRNA-605-5p (AUC = 0.76) were similarly altered in LOAD patients. Hence, these miRNAs may represent novel biomarkers for the development of both early- and late-onset AD (Mckeever et al., 2018).

Fernandes et al. (2018) found that the expression of ex-miRNA-21 was upregulated in CHME3 microglia derived from patients with AD. This work contributed to a deeper understanding of neuron–microglia communication and exosome-mediated neuroinflammation in AD and highlighted ex-miRNA-21 as a promising diagnostic biomarker for this disease (Fernandes et al., 2018).

## Ex-miRNAs as Biomarkers in PD

PD is a common neurodegenerative disease in the elderly, with an incidence rate of about 2% among people ≥65 years old (Zhang et al., 2005). The characteristic motor symptoms are static tremor, myotonia, bradykinesia, and postural balance disorder. With an increase in PD research, non-motor symptoms (NMS) of PD have gradually received closer attention. In particular, our understanding of PDD has greatly improved. Epidemiological survey results show that the incidence of PDD is 20% to 40%, which is seven times that of healthy people (Chaudhuri et al., 2006). Unlike AD, the diagnostic criteria of PDD do not emphasize memory impairment but place more emphasis on the impairment of the patients’ cognitive domain. Due to the complex clinical manifestations of this disease, there is an urgent need for PDD diagnostic biomarkers (Cammisuli et al., 2019). Presently, research on the differential expression of circulating ex-miRNAs as biomarkers has mainly focused on those with a PD diagnosis. Recently, studies have reported that, compared to healthy controls, differentially expressed ex-miRNAs can also be detected in the blood cells, plasma, serum, and CSF of patients with PD; some of these ex-miRNAs are helpful for the diagnosis of PD. Moreover, ex-miRNAs are abundant, have tissue specificity, and are highly stable. Therefore, ex-miRNAs may be suitable biomarkers for PD (Arshad et al., 2017).

Yao et al. (2018) explored the potential diagnostic value of plasma ex-miRNAs in patients with PD. The study included 52 patients with PD and 48 healthy controls. Compared to healthy controls, the plasma levels of ex-miRNA-331-5p were significantly higher, and those of ex-miRNA-505 was lower in PD patients (AUCs 0.849 and 0.898, respectively; Yao et al., 2018).

Cao et al. (2017) performed a study to investigate the expression of 24 candidate miRNAs and to evaluate their diagnostic value in patients with PD. In total, 109 patients with PD were enrolled and compared to 40 healthy controls. It was found that the serum levels of ex-miRNA-19b were significantly decreased (AUC = 0.753) while the levels of ex-miRNA-24 and ex-miRNA-195 were increased (AUC = 0.908 and 0.697, respectively) in PD patients, suggesting that the analysis of these ex-miRNAs in the serum could be helpful for the diagnosis of PD (Cao et al., 2017). Yang T. T. et al. (2018) also investigated the ability of serum exosome-derived miRNAs to distinguish subgroups of dementia patients (including 208 probable AD patients and 228 control subjects). The expression levels of ex-miRNA-135a, ex-miRNA-193b, and ex-miRNA-384 in the serum were analyzed. It was found that serum ex-miRNA-193b had the greatest diagnostic power (AUC = 0.996) in discriminating between AD and PDD (Yang T. T. et al., 2018). Additionally, serum ex-miRNA-137 levels were increased in patients with PD. It was also found that oxidation resistance 1 (OXR1) was down-regulated while miRNA-137 was up-regulated in PD patients. Jiang et al. (2019) suggested that miRNA-137 targeted OXR1 and negatively regulated its expression, contributing to oxidative stress injury in patients with PD. Barbagallo et al. (2020) detected 23 neurodegenerative disease-associated miRNAs in 139 participants, including patients with AD, PDD, VD, and VP. TaqMan reverse transcriptase–PCR data showed that serum ex-let-7d, ex-miRNA-22* (star represents that a mature miRNA is expressed from both the 5’-arm and the 3’-arm), ex-miRNA-23a, ex-miRNA-24, ex-miRNA-142-3p, and ex-miRNA-222 levels were increased in patients with PDD compared to healthy controls. These ex-miRNAs may discriminate dementia disorders in a non-invasive manner, thus improving the process of clinical diagnosis (Barbagallo et al., 2020).

Gui et al. (2015) developed a miRNA profiling strategy for ex-miRNAs isolated from CSF in the early stages of PD. They compared 47 PD patients to 27 healthy controls. They found that 16 ex-miRNAs were up-regulated and 11 ex-miRNAs were down-regulated in the CSF of patients with PD. Levels of ex-miRNA-1 (AUC = 0.920) and ex-miRNA-19b-3p (AUC = 0.705) were significantly decreased, while ex-miRNA-153 (AUC = 0.780), ex-miRNA-409-3p (AUC = 0.970), ex-miRNA-10a-5p (AUC = 0.90), and ex–let-7g-3p were significantly increased in the CSF of patients with PD (Gui et al., 2015). The development of reliable biomarkers for an accurate diagnosis in the earliest stages of PD has become an area of great interest in recent years. Dos Santos et al. (2018) analyzed CSF ex-miRNAs in a cross-sectional cohort comprising 40 early-stage PD patients and 40 well-matched healthy controls. They identified five CSF ex-miRNAs (miRNA-27a-3p, miRNA-125a-5p, miRNA-151a-3p, miRNA-423-5p, and let-7f-5p) with high sensitivity and specificity for differentiating PD patients from healthy controls (Dos Santos et al., 2018).

Based on the aforementioned studies, the use of circulating ex-miRNAs as biomarkers for early dementia diagnosis shows promise and is becoming a popular research topic (Supplementary Table S1). Currently, the most commonly studied ex–miRNAs have been mainly investigated for their utility in AD diagnosis, including ex-miRNA-342-3p, miRNA-125a-5p, miRNA-125b-5p, miRNA-23a-3p, miRNA-126-3p, miRNA-let-7i-5p, miRNA-151a, miRNA-132, miRNA-212, miRNA-223, miRNA-143-3p, miRNA-424-5p, miRNA-18b-5p, miRNA-3065-5p, miRNA-582-5p, miRNA-1306-5p, miRNA-15b-3p, miRNA-135a, miRNA-193b, miRNA-384, miRNA-22*, miRNA-23a, miRNA-29a, miRNA-125b, miRNA-9-5p, and miRNA-598. Among these, miRNA-342-3p is the most widely studied, and monitoring changes in miRNA-342-3p in peripheral fluids may be most helpful for the early diagnosis of AD. Besides, monitoring changes in ex-miRNAs may also help assess the extent of cognitive impairment and predicting the progression of the disease. For example, ex-miRNA-193b levels in patients with AD were lower than those in patients with MCI. ex-miRNA-16-5p, ex-miRNA-451a, and ex-miRNA-605-5p were found to be significantly increased, while the ex-miRNA-125b-5p level was decreased in AD patients. Moreover, ex-miRNA-135a, miRNA-193b, let-7d, miRNA-22*, miRNA-23a, miRNA-24, miRNA-142-3p, and miRNA-222 levels also have clinical value for the diagnosis of PDD, especially miRNA-193b. With the increase in research and the advancement of ex-miRNA detection technology, we look forward to the clinical use of circulating ex-miRNAs as diagnostic biomarkers of dementia.

## Ex-miRNAs as Biomarkers in Other Dementia Disorders

VD is the second most common dementia disorder and comprises a heterogeneous group of conditions with a wide range of clinical and neuropathological presentations. Vascular pathology in the brain impairs superior cortical processes, including planning, reasoning, and memory as impaired cerebral blood flow leads to irreversible secondary focal neuronal injury (O’brien and Thomas, 2015; Romay et al., 2019). The prevalence of VD in China is 1.1% to 3.0%, and the annual incidence is 5–9/1,000 people (Chen et al., 2016; Yang et al., 2017). Early diagnosis of VD is important for preventing the occurrence and development of dementia. Serum levels of ex-miRNA-135a were found to be increased and levels of ex-miRNA-193b were found to be decreased in patients with VD compared to healthy controls (Yang T. T. et al., 2018). Barbagallo et al. (2020) compared serum ex-miRNA expression in samples from 24 patients with VD and 30 healthy controls. They found that the levels of ex-miRNA-23a (AUC = 0.673), ex-miRNA-29a (AUC = 0.671), and ex-miRNA-130b (AUC = 0.683) were significantly decreased in patients with VD (Barbagallo et al., 2020).

DLB is a group of neurodegenerative diseases that overlap clinically and pathologically with PD and AD. DLB patients display fluctuating cognitive dysfunction, visual hallucinations, and Parkinson’s syndrome as clinical features, and Lewy bodies as a pathological feature (Jellinger, 2018). As stated above, plasma ex-miRNA-451a and ex-miRNA-21-5p were significantly decreased (AUC = 0.95 and 0.93, respectively) in patients with AD compared to DLB patients, which may help to improve the differential diagnosis of DLB vs. AD (Gámez-Valero et al., 2019).

FTD is a type of degenerative CNS disease and is characterized by latent onset, progressive behavioral abnormalities, and language disorder. Evidence of frontotemporal lobe atrophy and hypometabolism is visible upon the examination of images. The main histopathological manifestations are loss of neurons, eosinophilic swollen neurons, gliosis, spongy or vacuolated changes of surface nerve felt, and Pick bodies. Molecular biology studies have shown that FTD is a tau-related disease (Olney et al., 2017). Ex-miRNA expression studies have also been performed in patients with FTD. Schneider et al. (2018) assessed miRNA expression in a Genetic Frontotemporal Dementia Initiative cohort and in sporadic FTD patients (D’anca et al., 2019). In their study, CSF ex-miRNAs were isolated from 15 symptomatic, 23 pre-symptomatic and mutation carriers, and 11 healthy controls. CSF ex-miRNA-204-5p and ex-miRNA-632 were significantly decreased in symptomatic patients compared to pre-symptomatic mutation carriers (AUC = 0.89 and 0.91, respectively). Also, the levels of ex-miRNA-632 were significantly decreased in sporadic FTD patients compared to healthy controls (AUC = 0.90; Schneider et al., 2018).

## Conclusions and Prospects

Changes in the levels of miRNAs in peripheral body fluids are susceptible to various factors and cannot accurately reflect the progression of dementia (Song and Lee, 2015; Viegas et al., 2017). Several circulating ex-miRNAs, including ex-miRNA-342-3p and ex-miRNA-193b, may be used as potential biomarkers for AD/PDD diagnosis due to their stability. If ex-miRNAs are to become clinically significant dementia biomarkers, more extensive research is required. The accurate elucidation of specific ex-miRNAs that are abnormally expressed during the progression of dementia is important (Cha et al., 2019). Since large variations in the levels of abnormally expressed circulating ex-miRNAs are found in patients with dementia, large sample sizes are required to fully verify these miRNAs (Arena et al., 2017). Also, factors that affect the changes in expression of circulating ex-miRNAs, such as age, genetics, and environment, need to be considered in the future application of ex-miRNAs as diagnostic biomarkers for dementia (Frisoni et al., 2017). Ex-miRNAs may be used in the diagnosis of dementia, although traditional clinical detection methods should be carried out in parallel with ex-miRNA detection. With the continued development of technology and ongoing in-depth research, the advantages of ex-miRNAs as biomarkers for the progression of dementia will gradually emerge to provide a sufficient theoretical basis for the early detection and prevention of dementia.

## Author Contributions

This manuscript was primarily written by XD. Figures were produced by DZ. JN supervised the work. All authors contributed to the article and approved the submitted version.

## Conflict of Interest

The authors declare that the research was conducted in the absence of any commercial or financial relationships that could be construed as a potential conflict of interest.
