# Supplementary material for: Circulating Exosome microRNAs as Diagnostic Biomarkers of Dementia
Source: Front Aging Neurosci. 2020 Sep 8;12:580199. doi: 10.3389/fnagi.2020.580199 (PMC7506134; doi:10.3389/fnagi.2020.580199)
Supplement: Supplementary file 1 [file Table_1.DOCX]

**Table 1.** Circulating ex-miRNAs utility as biomarkers in dementia and neurodegenerative diseases

| **﻿Comparison** | **Sample size** | **Validated changes (AUC if applicable)** | **References** |
| --- | --- | --- | --- |
| ﻿AD versus CTRL | 35 AD, 35 CTRL | miRNA-342-3p | Lugli et al., 2015 |
| AD versus CTRL | 150 AD, 150 CTRL | miRNA-342-3p | Tan et al., 2014 |
| AD versus CTRL |  | miRNA-125a-5p, miRNA-342-3p, miRNA-125b-5p | Hinske et al., 2014 |
| AD versus CTRL | 97 AD, 97 CTRL | miRNA-342-3p, miRNA-125b-5p | Rani et al., 2017 |
| AD versus MCI | 51 AD, 43 MCI | miRNA-193b | Liu et al., 2014 |
| AD versus CTRL | 10 AD, 11 DLB, 11 CTRL | miRNA-23a-3p, miRNA-126-3p, miRNA-let-7i-5p, miRNA-151a | Gamez-Valero et al., 2019 |
| AD versus CTRL | 11 AD, 9 CTRL | miRNA-132 (AUC = 0.77), miRNA-212 (AUC = 0.84) | Cha et al., 2019 |
| AD versus CTRL | 32 AD, 16 CTRL | miRNA-223 (AUC = 0.875) | Wei et al., 2018 |
| AD versus CTRL | 51 AD, 23 CTRL | miRNA-143-3p, miRNA-424-5p, miRNA-18b-5p, miRNA-3065-5p, miRNA-582-5p, miRNA-1306-5p, miRNA-15b-3p | Cheng et al., 2015 |
| AD versus CTRL | 101 AD, 208 CTRL | miRNA-135a, miRNA-193b, miRNA-384 | Yang et al., 2018b |
| AD versus CTRL | 30 AD, 30 CTRL | miRNA-22*, miRNA-23a, miRNA-29a, miRNA-125b (AUC = 0.71) | Barbagallo et al., 2020 |
| AD versus CTRL | 10 AD, 10 CTRL | miRNA-9-5p, miRNA-598 | Riancho et al., 2017 |
| YOAD versus LOAD | 17 YO-AD, 13 LO-AD | miRNA-16-5p (AUC = 0.76), miRNA-451a (AUC = 0.95), miRNA-605-5p (AUC = 0.71), miRNA-125b-5p (AUC = 0.72) | McKeever et al., 2018 |
| PD versus CTRL | 54 PD, 48 CTRL | miRNA-331-5p (AUC = 0.849), miRNA-505 (AUC = 0.898) | Yao et al., 2018 |
| PDD versus CTRL | 30 PDD, 208 CTRL | miRNA-135a, miRNA-193b | Yang et al., 2018b |
| PD versus CTRL | 109 PD, 40 CTRL | miRNA-19b (AUC = 0.753), miRNA-24 (AUC = 0.908), miRNA-195 (AUC = 0.697) | Cao et al., 2017 |
| PD versus AD | 101 AD, 30 PDD | miRNA-193b (AUC = 0.996) | Yang et al., 2018b |
| PDD versus CTRL | 30 PDD, 30 CTRL | let-7d, miRNA-22*, miRNA-23a, miRNA-24, miRNA-142-3p, miRNA-222 | Barbagallo et al., 2020 |
| PD versus CTRL | 47 PD, 27 CTRL | miRNA-1 (AUC = 0.920), miRNA-19b-3p (AUC = 0.705), miRNA-153 (AUC = 0.780), miRNA-409-3p (AUC = 0.970), miRNA-10a-5p (AUC = 0.90) | Gui et al., 2015 |
| PD versus CTRL | 40 PD, 40 CTRL | miRNA-27a-3p, miRNA-125a-5p, miRNA-151a-3p, miRNA-423-5p, let-7f-5p | Dos Santos et al., 2018 |
| VD versus CTRL | 24 VD, 30 CTRL | miRNA-23a (AUC = 0.673), miRNA-29a (AUC = 0.671), miRNA-130b (AUC = 0.683) | Barbagallo et al., 2020 |
| DLB versus AD | 11 DLB, 10 AD | miRNA-21-5p (AUC = 0.93), miRNA-451a (AUC = 0.95) | Gamez-Valero et al., 2019 |
| FTD versus CTRL | 38 FTD, 11 CTRL | miRNA-204-5p (AUC = 0.89), miRNA-632 (AUC = 0.91) | Schneider et al., 2018 |

﻿AUC, area under the curve; AD, Alzheimer’s disease; CTRL, control; DLB, dementia with Lewy body; FTD, frontotemporal dementia; LOAD, late onset AD; MCI, mild cognitive impairment; PD, Parkinson’s disease; PDD, Parkinson’s disease dementia; VD, vascular dementia; YOAD, young onset AD.
